# Symptomatology and Coping Resources Predict Self-Care Behaviors in Middle to Older Age Patients with Heart Failure

**DOI:** 10.1155/2015/840240

**Published:** 2015-11-05

**Authors:** Lucinda J. Graven, Joan S. Grant, Glenna Gordon

**Affiliations:** ^1^Florida State University College of Nursing, 98 Varsity Way, Duxbury Hall, Tallahassee, FL 32306-4310, USA; ^2^University of Alabama at Birmingham School of Nursing, 1720 2nd Avenue South, Birmingham, AL 35294-1210, USA

## Abstract

*Background.* Symptoms of heart failure (HF) and coping resources, such as social support and social problem-solving, may influence self-care behaviors. Research regarding the influence of HF symptomatology characteristics and components of social support and social problem-solving on self-care is limited.* Objective.* To identify predictors of HF self-care behaviors using characteristics of HF symptomatology, components of social support and social problem-solving, and demographic and clinical factors.* Methods.* Using a cross-sectional, correlational predictive design, a convenience sample (*N* = 201) of outpatients with HF answered self-report surveys. Multiple linear regression with stepwise variable selection was conducted.* Results.* Six predictors of HF self-care were identified: race, symptom frequency, symptom-related interference with enjoyment of life, New York Heart Association Class HF, rational problem-solving style, and social network (*β* = 34.265, *R* ^2^ = 0.19, *P* = 0.001).* Conclusions.* Assessing the influence of race on self-care behaviors in middle to older age patients with HF is important. Clinical assessment that focuses on symptom frequency, symptom-related interference with enjoyment of life, and HF Class might also impact self-care behaviors in this population. Rational problem-solving skills used and evaluation of the size of and satisfaction with one's social network may be appropriate when assessing self-care.

## 1. Introduction

Heart failure (HF) remains a significant burden on the health care industry in the United States. Each year about 870,000 new cases are diagnosed in the United States, and currently, an estimated 5.7 million Americans suffer from this disease. The mortality rate remains high, with one in five individuals dying within five years of diagnosis [1]. Additionally, HF remains one of the top diagnoses for hospital readmissions [2, 3], with total costs for HF treatment exceeding 30 billion dollars [1]. In fact, HF is the most common diagnosis for hospitalized patients 65 years and older [4]. Previous research suggests that patients who maintain self-care behaviors, such as adhering to treatment regimen, have improved outcomes, including fewer hospital readmissions and improved survival [5]. Therefore, improving self-care among patients with HF is vital in influencing adverse events such as frequent hospitalizations and mortality.

Self-care involves an active process that includes physical and decision-making processes [6, 7]. Physical activities are performed to reduce morbidity and preserve physiologic stability [6]. These physical activities are disease-specific and include adherence to medication, dietary and fluid restrictions, and regular exercise [7]. Decision-making processes used by individuals with HF include recognizing health changes, evaluating health status and decisions to take action, implementing potential solutions to address health-related issues, and evaluating the effectiveness of solutions [8]. Certain individual (e.g., race, gender, age, marital status, income, educational level, and number of people who live in a household) and clinical (e.g., NYHA Class HF, length of time since diagnosis) characteristics may influence self-care indirectly by impacting other factors (i.e., cognitive, psychosocial, physical, and sociocultural) which influence self-care [9–13].

Living with HF is stressful, with symptomatology increasing as HF progresses [7, 14]. Characteristics of HF symptoms may impact the ability to perform self-care activities and require utilization of coping resources, such as social support and social problem-solving. Studies suggest patients experiencing severe symptoms have greater difficulty recognizing and responding to increased symptoms [15, 16]. Thus, many patients rely on caregivers for assistance with symptom assessment and management [17]. Likewise, patients experiencing increased symptom frequency and severity are particularly vulnerable to dependency upon others for assistance with self-care [18]. Other research contradicts, suggesting more frequent symptoms of HF are associated with better self-care [19]. Nonetheless, studies examining the characteristics of HF symptoms (i.e., frequency, severity, and degree of symptom-related interference with activity and enjoyment of life) are limited [20]. Thus, how symptom characteristics influence self-care behaviors in HF is relatively unknown, requiring more research.

The relationship between social support and self-care in HF patients has been widely studied [21–23]. While research indicates increased levels of social support are associated with better self-care [23, 24], the majority of these studies have investigated only perceived support [23]. However, little is known about which type of perceived support (i.e., belonging, tangible, or appraisal) is most beneficial in influencing self-care in patients with HF. Similarly, few studies have investigated the influence of social networks (e.g., family and friends), despite research suggesting this type of support positively influences self-care in those with HF [25]. Hence, more research is needed to examine how both types of support influence self-care behaviors in this population.

Social problem-solving may also influence self-care behaviors in patients with HF. Social problem-solving is defined as problem-solving in a real world environment and involves problem orientation and problem-solving style [26]. Published studies examining social problem-solving in those with HF are limited [27]; however, studies examining patients with diabetes mellitus suggest that the manner in which individuals solve problems directly influences self-care behaviors, specifically self-management [10, 28, 29]. Furthermore, findings suggest that impulsivity/carelessness and avoidant problem-solving styles are associated with worse self-care in diabetic patients [10]. To date, no published studies have examined the influence of problem orientation and problem-solving styles on HF self-care. Information related to these relationships could aid in clinical management and patient education for those with HF.

## 2. Conceptual Framework

This study was guided by concepts from the theory of stress and coping [30], which describes how individuals adapt psychologically to stressful situations. Stress is a relationship between individuals and their environment that when appraised is determined to exceed personal resources and serve as a threat to well-being. Coping is a process utilized by individuals to manage a situation that is stressful and emotions that accompany the situation. Individual and clinical characteristics may potentially influence disease-related stressors and coping resources, which affect disease-related outcomes [30].

**Figure 1:**
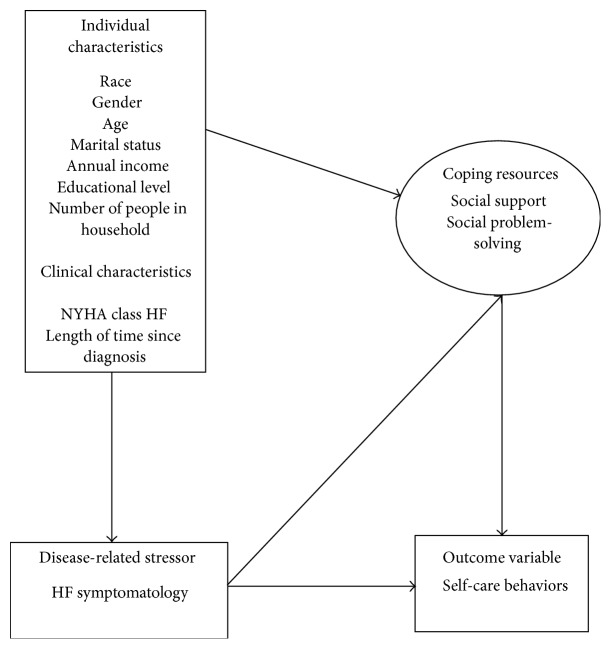
Conceptual model of study variables based on Lazarus and Folkman's Theory of Stress, Appraisal, and Coping [29].* Notes.* NYHA = New York Heart Association, HF = heart failure.

In this study, HF symptom frequency, severity, and degree of symptom-related interference with physical activity and enjoyment of life are viewed as potential stressors that influence self-care behaviors in individuals living with HF. Social support and social problem-solving are potential coping resources used to manage HF symptomatology and improve HF self-care behaviors (Figure 1). Hence, the purpose of this study was to identify predictors of self-care behaviors from among characteristics of HF symptomatology (i.e., frequency, severity, and degree of symptom-related interference with physical activity and enjoyment of life), social support (i.e., belonging, tangible, and appraisal support; social network), and social problem-solving (i.e., positive and negative problem orientation; rational, impulsivity/carelessness, and avoidance problem-solving styles), in addition to individual and clinical characteristics in patients with HF of age 55 years and older.

## 3. Methods

### 3.1. Study Design and Participants

This study used a cross-sectional, correlational, predictive design to investigate predictors of self-care behaviors in a convenience sample of 201 outpatients with HF. A power analysis for multiple linear regression was conducted, using a medium effect size, 80% power, significance level of 0.05, and variables identified in Tables 1 and 2 [31, 32]. The minimum desired sample size was 166. Patients with a diagnosis of HF, age 55 years and older, who resided in an outpatient setting were included in the study. The age range for inclusion in this study was limited to specifically examine predictors of self-care behaviors in middle-older age adults with HF. Therefore, the Telephone Interview for Cognitive Status (TICS) [33] was used to screen for the potential of cognitive impairment. Individuals with a score of ≤30 on the TICS were excluded from study participation due to the possibility of impaired cognition.

### 3.2. Setting and Procedure

After institutional review board approval, potential participants were recruited using letters and flyers from three hospital-affiliated outpatient offices in North Florida. Those patients interested in the study contacted the primary investigator and underwent clinical and cognitive screening for inclusion over the telephone. Following telephone screening for inclusion and exclusion, potential participants who met study criteria were then scheduled for an individual interview at their physician's office. After obtaining informed consent, participants were interviewed using a set of self-report surveys presented in random order. Interviews were conducted in a private location within the physician's office. No incentives for participation were offered.

### 3.3. Measures

A researcher-developed sociodemographic and clinical survey was used for clinical screening and to obtain information on participant characteristics. Clinical information was gathered via self-report and included questions regarding the length of time since HF diagnosis and the severity of HF based upon the New York Heart Association (NYHA) Classification for HF [34]. Cognitive screening was conducted using the 11-item TICS, which assesses orientation, attention, language, learning, and memory. The highest score is 41, with ≤30 suggesting potential for impaired cognition. Prior research supports its validity and reliability [33]. A total of five instruments were used to measure study variables.

#### 3.3.1. Heart Failure Symptoms

The 14-item Heart Failure Symptom Survey (HFSS) [20] was used to measure four characteristics of HF symptomatology: frequency, severity, degree of interference with physical activity, and degree of interference with enjoyment of life based upon the last week. Participants rate each symptom using an 11-point scale (i.e., 0–10). Higher scores on a particular domain suggest more frequent and severe symptoms and greater interference with physical activity and enjoyment of life, respectively [20]. Previous studies support the validity [20] and reliability (*α* > 0.80) of each domain [17]. In this study, Cronbach's alphas were adequate for all domains, including frequency (*α* = 0.795), severity (*α* = 0.857), interference with physical activity (*α* = 0.853), and interference with enjoyment of life (*α* = 0.878).

#### 3.3.2. Social Support

Social support is a multifaceted concept and involves both perceived support and social network. Thus, in order to fully examine the concept, we used two measures of social support. The Interpersonal Support Evaluation List-12 (ISEL-12) [35] was used to measure three types of perceived support (i.e., belonging, tangible, and appraisal support). Scores range from 0 to 36, with higher scores indicating a higher perception of available support with regard to the particular subscale [36]. Prior studies support adequate validity [26] and reliability (*α* = 0.94) [27]. Internal consistency, using Cronbach's alpha, was adequate for all subscales (*α* > 0.70) in this study.

The Graven and Grant Social Network Survey (GGSNS) [27] is a 12-item survey that was used to measure social network. Participants identify the number of people in their life who provide assistance and support, as well as rating their satisfaction with provided support. Scores range from 12 to 84, with higher scores suggesting higher levels of actual support. Content and construct validity, as well as reliability (*α* = 0.93), were established in a previous study [27]. Cronbach's alpha was 0.89 in the current study.

#### 3.3.3. Social Problem-Solving

Social problem-solving was measured using the Social Problem-Solving Inventory Revised-Short (SPSIR-S) [36]. This 25-item survey represents both positive and negative problem orientation, as well as three problem-solving styles (i.e., rational, impulsivity/carelessness, and avoidance). Scores for each domain range from 0 to 20. Higher scores represent more of the respective domain. Adequate validity [36] and reliability have been reported previously using a total score for adaptive and maladaptive items (*α* = 0.86 and 0.77, resp.) [27]. With the exception of the positive problem orientation subscale (*α* = 0.672), all other subscales were internally consistent with Cronbach's alphas greater than 0.80.

#### 3.3.4. Heart Failure Self-Care Behaviors

The European Heart Failure Self-care Behavior Scale-9 (EHFScBS-9) was used to measure self-care behaviors related to HF. This survey identifies nine activities specific to HF self-care and participants rate their level of agreement on a 5-point scale. Total score ranges from 9 to 45, with higher scores indicating worse self-care behaviors. Prior research has supported its validity and reliability (*α* = 0.87) [37] and in this study Cronbach's alpha was 0.67.

## 4. Data Analysis

Data were analyzed using the Statistical Package for the Social Sciences (SPSS) version 20. Descriptive statistics (e.g., mean, standard deviation, percent, and frequencies) were conducted to examine sample characteristics and scores on all study variables. Internal consistency was assessed on all study scales, using Cronbach's alpha. Multiple linear regression with true stepwise variable selection was used to examine the impact of the study variables, using subscales versus total scores when applicable, on self-care behaviors, taking into account the potential influence of the other variables in the model. The model was fit with the total score on the EHFScBS-9 as the dependent variable. The addition/removal criteria in SPSS were used for stepwise variable selection, including a probability of *F* of 0.05 for addition and 0.10 for removal from the model. Individual and clinical characteristics included in the initial model were gender, marital status, age, race, highest level of education attained, number of people in the household, annual income, NYHA Class HF, and length of time since HF diagnosis. Additionally, the following variables were included as predictor variables: frequency and severity of HF symptoms; degree of symptom-related interference with physical activity and enjoyment of life; appraisal, tangible, and belonging support; positive and negative problem orientation; and rational, impulsivity/carelessness, and avoidance problem-solving styles. Underlying assumptions for multiple regression were examined.

## 5. Results

### 5.1. Sample Characteristics and Descriptive Analysis

Telephone screening was conducted on 205 participants, with one scoring less than 30 on the TICS and three which failed to follow up for the scheduled interview. Thus, a total of 201 participants were included in the study (Table 1). Participants were predominantly nonminority (86.1%) males (62.7%). The average age of participants was 72.6 (SD, 8.9). Most participants had NYHA Class II HF. The average score on the EHFScBS-9 was 25.65, indicating that the majority of the participants reported poor self-care behaviors. The majority of participants were not experiencing frequent (1.98 [SD, 1.64]) nor severe (1.69 [SD, 1.62]) symptoms of HF. There was little symptom-related interference with physical activity (1.20 [SD, 1.17]) and enjoyment of life (SD, 1.14 [1.58]) reported. On average, participants reported a larger social network (56.46 [SD, 18.73]) and above average appraisal (9.74 [SD, 3.05]), tangible (10.30 [SD, 2.47]), and belonging (9.05 [SD, 3.02]) support. Likewise, greater-than-average scores were noted on all subscales of the SPSIR-S (Table 2).

### 5.2. Predictors of Self-Care Behaviors

Using a level of significance set at 0.05, a total of six models were tested (Table 3). The final model identified six significant predictors of self-care behaviors, including race (*β* = −4.362; *P* = 0.002), frequency of HF symptoms (*β* = 1.888; *P* = 0.002), HF symptom-related degree of interference with enjoyment of life (*β* = −1.394; *P* = 0.023), NYHA Class HF (*β* = 1.180; *P* = 0.029), rational problem-solving (*β* = −0.247; *P* = 0.025), and social network (*β* = −0.095; *P* ≤ 0.001). The overall significance of the model was *P* < 0.001 (*β* = 34.265), with an adjusted *R* ^2^ for the final model of 0.19, indicating that the set of predictors accounted for approximately 19% of the variance in self-care (Table 4). Underlying assumptions for multiple linear regression were evaluated using (1) a normal probability plot of residuals, (2) a scatterplot of predicted values versus residuals, (3) collinearity statistics, and (4) reliability of study variables. Based upon these, assumptions of multiple regression were not violated and no issues with multicollinearity were noted (all tolerance and variance inflation factor values in the final model were within acceptable ranges [tolerance > 0.1; VIF < 10]).

## 6. Discussion

In this study, we focused on predictors of self-care behaviors in middle to older age patients with HF. Regression analyses revealed six predictors of HF self-care behaviors, with race contributing the most to self-care behaviors in this study. Our findings were inconsistent with that of prior research. While few studies have investigated race as a predictor of self-care, Davis and colleagues [38] found that nonblacks had higher self-care maintenance scores as compared to blacks. This is inconsistent with our findings, which indicated minorities have better self-care, scoring, on average, 4.37 points lower than nonminorities when controlling for the other variables. This finding was surprising, given that minority race has been associated with several negative prognostics of self-care, including lower health literacy [39], decreased quality of health care [40], and decreased knowledge of HF [38]. In this sample, minorities reported a larger social network as compared to nonminorities (58.04, SD 19.61, versus 56.20, SD 18.64), perhaps contributing to this finding. Prior studies investigating support in minorities have suggested that social network is a significant stress buffer [41] and may have influenced self-care in this study, warranting further research.

Issues related to symptomatology also predicted self-care behaviors in this sample. Frequency of HF symptoms was found to contribute more than symptom-related interference with enjoyment of life to self-care behaviors. At this time, research is limited investigating whether symptom frequency predicts self-care in those with HF. Research does, however, suggest that progression of HF symptoms influences an individuals' ability to sustain adequate self-care behaviors [42, 43]. No published studies, to date, have examined symptom-related interference with enjoyment of life as a predictor of self-care behaviors in individuals with HF. Our findings suggest that symptom-related interference with enjoyment of life also predicts self-care behaviors. In fact, better self-care was noted in those patients whose symptoms interfered with their enjoyment of life, suggesting that individuals with HF may not report symptoms or seek treatment until symptoms significantly interfere with their enjoyment of life. Thus, findings illustrate importance of a thorough symptom assessment at each health care visit, examining not only frequency and severity of symptoms, but also how symptoms impact daily life and leisure activities.

In this study, approximately 50% of patients reported poor self-care behaviors, which is consistent with prior studies [38, 44]. Patients with NYHA Class IV HF reported worse self-care behaviors than those with NYHA Class I–III HF. Additionally, a higher severity of HF, based upon NYHA Class, predicted worse self-care behaviors, when controlling for other variables in the model. Findings in the literature related to the relationship between HF severity and self-care are inconsistent. While some studies indicate patients experiencing more symptoms and functional impairment practice better self-care [38, 45], qualitative research suggests that functional limitations and severe symptoms are actually barriers to effective self-care [42]. Our findings support that of Riegel and Carlson [42] by implying that disease severity and subsequent functional limitations may actually hinder self-care behaviors. Though more research is needed to investigate the relationship between disease severity and self-care, our results do provide a target for clinical assessment and management, as well as patient education.

An important finding in this study was that rational problem-solving, a coping resource, contributed to better self-care behaviors. In this study, patients utilizing rational problem-solving strategies, such as planful problem-solving, active coping, and seeking social support, had better self-care than patients who used other strategies such as avoidance, denial, and behavioral/mental disengagement [36]. To our knowledge, previous published studies have not studied rational problem-solving and self-care in individuals with HF. However, rational problem-solving and the development of problem-solving skills have been examined in diabetic patients, with use of these skills found to be independently associated with disease-specific self-management behaviors, such as diet and exercise [29]. Use of rational problem-solving skills, including problem identification and goal setting, has also been identified as a central concept involved in self-efficacy in those with chronic illnesses [46]. Findings of this study provide potential for intervention development; still, more research is needed in this area to provide support of these findings in patients with HF.

The availability of and satisfaction with one's social network contributed the least to self-care behaviors, which may be due to other variables not examined in this study (e.g., family relationships). In this study, patients who reported higher social network scores had better self-care than those patients who reported lower social network scores. While studies investigating social network are limited [12, 24], this finding supports previous work, which suggests that a larger social network is related to HF self-care management and confidence [24], as well as fewer hospital readmissions [12]. While the influence of family support has been well documented in the literature [7, 17, 25], this study did not limit the investigation of social network to family but instead included anyone who might be involved in the provision of support and who may aid in effective coping (e.g., friends, neighbors, and church/social club members).

This study provides important information related to predictors of self-care behaviors and the influence of symptomatology and coping resources. Yet the low adjusted *R* ^2^ suggests that an unmeasured variable may play an important role in the model and this finding should be considered in future studies. For example, an important component not measured in this study is health literacy. Previous research, however, is not consistent with some studies identifying it as a potential barrier to effective HF self-care [7] and others [47] finding no relationship between health literacy and self-care in patients with HF. Additionally, there may be other unmeasured variables that could potentially play a role in the prediction of self-care behaviors, such as comorbidities and cognitive status, warranting further research in this area.

To our knowledge this study is the one of the first to examine the subcomponents of HF severity and social problem-solving in patients with HF, providing information useful for intervention development. The inclusion of social network, in addition to subcomponents of perceived social support, allows researchers to examine which component of social support is the stronger predictor of self-care, as most studies have previously only regressed perceived social support on self-care behaviors using a total score [21, 22, 24]. While this study provides several target areas for clinical management, limitations exist. The majority of participants were nonminority men with NYHA Class II HF, limiting generalizability of findings to similar individuals. In addition, this study included only patients 55 years and older, limiting examination of these variables in a younger population of HF patients. The cross-sectional nature of this study restricts our understanding of the influence of these variables on HF self-care and does not provide information regarding how these relationships may change over time as HF progresses. Also, little variation with regard to the characteristics of HF symptomatology existed within the sample and may have contributed to measurement error. Finally, the relatively asymptomatic nature of most participants may have impacted the reliability of the EHFScBS-9, as many of the questions on this instrument were more appropriate for patients who were symptomatic.

## 7. Conclusions

Middle to older age patients with HF are susceptible to adverse outcomes related to poor self-care. This study advances our understanding of how symptomatology and coping resources may influence self-care behaviors in patients with HF, taking into account the limitations of this study. Assessing the influence of race on self-care behaviors in middle to older age individuals with HF is important. Monitoring symptom frequency, degree of symptom-related inference with enjoyment of life, and class of heart failure at clinical visits is recommended, as patients may not complain of symptoms until symptoms impact their enjoyment of life. Similarly, appraisal and teaching of rational problem-solving skills to address heart-related issues may be useful in enhancing self-care behaviors in this population. Evaluating the size of and satisfaction with one's social network is appropriate when assessing self-care, with referral to community resources, if needed.

## Figures and Tables

**Table 1:** Sample characteristics and mean self-care score.

	*n*	%	Mean score on EHFScBS-9 ± SD
Gender			
Male	126	62.7	26 ± 7
Female	75	37.3	25 ± 8
Age group			
55–64 years	37	18.4	25 ± 8
65–74 years	81	40.3	25 ± 8
75–84 years	60	29.9	26 ± 7
Older than 85 years	23	11.4	29 ± 8
Marital status			
Married or living with significant other	117	58.2	25 ± 7
Single, divorced, widowed	84	41.8%	26 ± 8
Race			
Nonminority	173	86.1	26 ± 8
Minority	28	13.9	22 ± 6
Annual income			
Under $30,000	34	16.9	25 ± 7
$30,000–$50,000	66	32.8	26 ± 8
$50,000–$75,000	61	30.3	24 ± 8
$75,000–$100,000	34	16.9	27 ± 7
Over $100,000	4	2.0	28 ± 12
Highest level of education			
No HS diploma	22	10.9	24 ± 7
HS diploma	50	24.9	26 ± 7
Some college or certification course	46	22.9	25 ± 8
Bachelor's degree	63	31.3	26 ± 8
Graduate degree	20	10.0	25 ± 9
NYHA HF Classification			
I	39	19.4	23 ± 8
II	95	47.3	26 ± 8
III	23	11.4	25 ± 6
IV	44	21.9	28 ± 7
Length of time since HF diagnosis			
<1 year	19	9.5	24 ± 7
1–5 years	54	26.9	25 ± 7
5–10 years	58	28.9	27 ± 8
10–15 years	34	16.9	25 ± 9
>15 years	36	17.9	27 ± 7

Notes: HS: high school; NYHA: New York Heart Association; HF: heart failure.

**Table 2:** Study instruments: descriptive statistics.

Scale	Subscales used	Possible range	Actual range	Mean (SD)
Heart Failure Symptom Survey (HFSS)	Frequency of heart failure symptoms	0–10	0–8	1.98 (1.64)
	Severity of heart failure symptoms	0–10	0–7.86	1.69 (1.62)
	Heart failure symptom-related degree of interference with physical activity	0–10	0–7.43	1.20 (1.47)
	Heart failure symptom-related degree of interference with enjoyment of life	0–10	0–7.64	1.14 (1.58)

Grant and Graven Social Network Survey (GGSNS)	Total scale	12–84	12–84	56.46 (18.73)

Interpersonal Support Evaluation List-12 (ISEL-12)	Appraisal support	0–12	0–12	9.74 (3.05)
	Tangible support	0–12	0–12	10.30 (2.47)
	Belonging support	0–12	0–12	9.05 (3.02)

Social Problem-Solving Inventory Revised-Short (SPSIR-S)	Positive problem orientation (PPO)	0–20	5–20	14.27 (3.80)
	Rational problem-solving (RPS)	0–20	0–20	12.95 (4.50)
	Negative problem orientation (NPO)	0–20	0–20	12.89 (6.51)
	Impulsivity/carelessness style (ICS)	0–20	0–20	12.68 (5.98)
	Avoidance style (AS)	0–20	0–20	12.87 (6.39)

European Heart Failure Self-care Behavior Scale-9 (EHFScBS-9)	Total scale (response variable)	9–45	9–45	25.65 (7.54)

**Table 3:** Model summary.

Model	*R*	*R* square	Adj. *R* square	Std. error of the estimate	Change statistics				
					*R* square change	*F* change	df1	df2	Sig. *F* change
1	0.273	0.074	0.070	7.280	0.074	16.002	1	199	0.000
2	0.338	0.114	0.105	7.139	0.040	8.890	1	198	0.003
3	0.392	0.154	0.141	6.996	0.039	9.191	1	197	0.003
4	0.417	0.174	0.157	6.931	0.020	4.744	1	196	0.031
5	0.436	0.190	0.169	6.879	0.016	3.973	1	195	0.048
6	0.458	0.210	0.185	6.812	0.020	4.832	1	194	0.029

Notes: HF: heart failure; RPS: rational problem-solving.

(1) Predictors: (Constant), Total Score for Social Network.

(2) Predictors: (Constant), Total Score for Social Network, frequency of HF symptoms.

(3) Predictors: (Constant), Total Score for Social Network, frequency of HF symptoms, Race for Analysis.

(4) Predictors: (Constant), Total Score for Social Network, frequency of HF symptoms, Race for Analysis, HF symptom-related degree of interference with enjoyment of life.

(5) Predictors: (Constant), Total Score for Social Network, frequency of HF symptoms, Race for Analysis, HF symptom-related degree of interference with enjoyment of life, Problem-Solving Subscale (RPS).

(6) Predictors: (Constant), Total Score for Social Network, frequency of HF symptoms, Race for Analysis, HF symptom-related degree of interference with enjoyment of life, Problem-Solving Subscale (RPS), Heart Failure Class.

**Table 4:** Final multiple linear regression analysis using EHFScBS-9 (total score) as outcome.

Variable	Estimate	Standard error	*P* value
Intercept	34.265	2.683	<0.001
Social network	−0.095	0.026	<0.001
Frequency of heart failure symptoms	1.888	0.590	0.002
Race (ref.: minority versus nonminority)	−4.362	1.401	0.002
Degree of interference with enjoyment of life	−1.394	0.610	0.023
Rational problem-solving	−0.247	0.109	0.025
NYHA HF Class (ref.: IV versus I–III)	1.180	0.537	0.029

Notes: NYHA: New York Heart Association; HF: heart failure.
